# The Influence of Dosing Method and Material Characteristics of Superabsorbent Polymers (SAP) on the Effectiveness of the Concrete Internal Curing

**DOI:** 10.3390/ma11091600

**Published:** 2018-09-03

**Authors:** Piotr P. Woyciechowski, Maciej Kalinowski

**Affiliations:** Department of Building Materials Engineering, Warsaw University of Technology, 00-637 Warsaw, Poland

**Keywords:** cement concrete, internal curing, superabsorbent polymers, shrinkage, absorption, desorption

## Abstract

This paper examines the influence of dosing method and material characteristic of superabsorbent polymers (SAP) used for internal curing, on the selected concrete properties. A new method of introducing SAP into the concrete mix and its impact on the shrinkage and compressive strength of concrete was studied. It was shown that the method of dosing SAP to the concrete mix and the differences in the properties of the tested SAPs have a significant impact on the course of changes of selected properties of the tested concrete composites. In order to compare tested series with each other and with other published results on the subject, a new method of including SAP mass content in the concrete mix, as a percentage of absorbed mixing water, was presented. The effectiveness of internal curing using different types of SAP under different dosing methods was presented as a percentage difference in tested concrete properties between modified series and reference series.

## 1. Introduction

Superabsorbent polymers (SAP) can be described as cross-linked hydrogel networks [1,2,3]. SAP owe its ability to absorb water up to hundreds of times higher than their own mass to high osmotic pressure caused by the accumulation of trapped ions in the SAP structure [4,5,6]. The absorbed water causes the SAP to swell, thereby removing the ions from each other, which results in the reduction of the osmotic pressure [7,8,9,10].

Assuming such a model of an interaction within SAP, its their absorption capacity is limited not only by lowering the osmotic pressure due to water absorption, but also by the influence of external pressures resulting from the change of the SAP volume [3,4] and the alkalinity of the environment in which the absorption takes place [11]. In the case of a loss of balance between osmotic pressure within the SAP saturated with water, the internal stresses of concrete, and the influence of capillary forces in the porous medium, SAP is able to reduce its volume, i.e., to desorb water [12,13]. In concrete technology this effect is used for internal curing [14,15,16,17,18].

SAP as a component of concrete is difficult to classify using the current criteria, due to the non-standard parameters that characterize its role in the concrete mix. According to the quantitative criterion, SAP may be included in the group of concrete admixtures according to EN 206 standard, because its dosage is usually 0.3–0.6% of binder mass [1]. Introducing SAP into the concrete mix affects absorption of a portion of the mixing water, significantly exceeding the mass and volume of the added SAP. In the case of SAP that has been previously added to the mixing water, the amount of which has been set at, for example, 0.2% of the binder mass, and of which tap water absorption capacity is about 100 g/g, the mass ratio of the saturated SAP related to the cement mass changes about 100 times. If SAP pre-saturated in tap water is introduced as a component of the concrete composite, in quantitative terms it can be treated as an additive. The role of SAP in concrete is also important. It does not modify the properties of individual components of the concrete mix, as is the case with most admixtures, but improves the properties of the concrete composite through internal water curing. Due to the addition of SAP into the concrete mix, an additional gel phase in the form of an SAP saturated with water forms, which affects the pore phase distribution after the desorption of water [19].

The published research on SAP in concrete technology focuses primarily on the assessment of the impact of various types of SAP on the mechanical properties and durability of concrete composites, as well as on the properties of concrete mixes [5,6,7,8,9,10]. Relatively little research is devoted to the technology of making concrete mixes with the addition of SAP, including the problem of pre-saturating SAP with tap water before adding it to the concrete mix. In the literature [20], the majority of studies include the addition of unsaturated SAP with other dry components of a concrete mix. The mixing water and sometimes a superplasticizer are then added. With this methodology of making concrete mix, where the activation of the SAP (the process of reaching maximal saturation in any given environment) takes place at the stage of mixing all components, the absorption capacity of 8–30 g/g of the SAP is determined as its absorption potential in a highly alkaline environment [20]. It is about 10 times less than in a tap water environment. The reduced absorption capacity in the case of dosing the non-activated SAP into the mix results in over-estimation of the mass content of SAP in relation to the mass of cement required to absorb the designed amount of mixing water. Despite the fact that in some of the tests carried out, SAP content in the concrete mix was as much as 1% of the cement mass, the percentage of absorbed mixing water was an order of magnitude smaller than in the case of adding SAP pre-saturated with tap water to the rest of concrete mix components [21].

**Figure 1:**
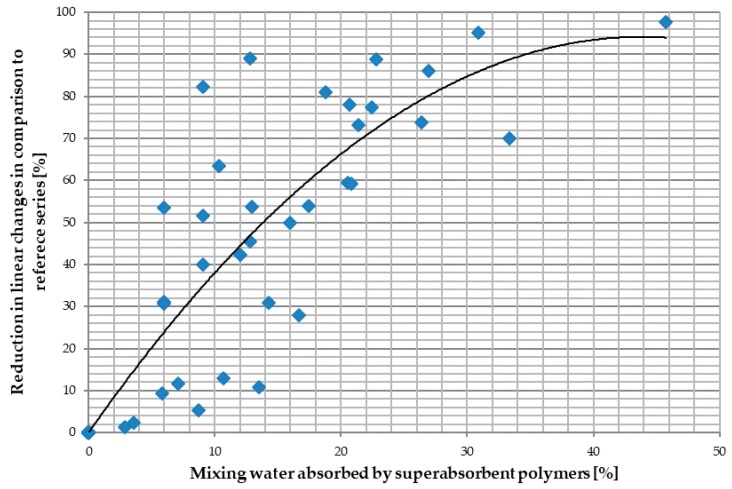
Reduction of linear changes in relation to the reference series not modified with superabsorbent polymers (SAP) and the percentage of the water absorbed by SAP commonly used in the published studies method of dosing SAP to the concrete mix The graph was prepared by the authors based on the published research results [20,26,27,28,29].

The issue of SAP’s adequate preparation and insertion method into the concrete mix is, in the authors’ opinion, crucial in the context of obtaining the optimal internal curing effect in relation to the basic characteristics of concrete, including primarily autogenous shrinkage and mechanical features. Autogenous shrinkage is one of the parameters that reduce the durability of concrete composites with low water-binder ratio [16,22,23,24]. Analyzing the published research results, one can observe a significant reduction of shrinkage deformations with the increase in the amount of mixing water absorbed by SAP, reaching almost 100%, as seen in Figure 1. It is also possible to find examples of reduction exceeding 100% (swelling of the material), however, this is mainly the case when SAP is used in concrete composites with low w/b ratio [25] or in blast furnace slag as a part of the binder [26].

Despite the fact that the use of SAP as an internal curing agent is aimed at improving the durability of a concrete composite, such as reduction of linear changes, SAP can also have an influence on the mechanical parameters of concrete composites. Due to the theoretical mechanism of SAP activation, involving the absorption of mixing water from a concrete mix by a previously unsaturated SAP, changes in the course of hydration [29,30,31] or changes in the pore phase distribution in hardened concrete can be expected [21,27,28,32,33,34].

Analyzing the published research results, the decrease in compressive strength is visible as the proportion of water absorbed in the total amount of mixing water increases [27,32,33,35,36,37,38]. The reason behind this behavior of the concrete composite is the uneven distribution of SAP in the concrete mix. It is the result of the commonly used method of dosing the non-activated SAP to the dry components of the concrete mix [20]. Due to its electrochemical properties, SAP contributes to the formation of a quasi-pore network after fulfilling its role as an internal curing factor. These pores, referred to as defects [20,30,31], foster the disruption of the structure of the cement matrix, leading to a reduction in the strength of the concrete composite. It has been proven that even distribution of SAP within concrete mix leads to the increase in average compressive strength in UHPC (Ultra High Performance Concrete) [19].

In the vast majority of tests carried out, the methodology known from other methods of internal concrete curing is adopted. It consists of introducing an additional volume of water to the concrete mix, which corresponds with the absorption capacity of the applied SAP mass. This fact influences the method of calculating the water-binder ratio. Usually, with respect to concrete composites with SAP addition, three variants of this coefficient are distinguished: (w/c)_tot_—total water-binder ratio, (w_e_/c)—ratio of entrained water to the mass of the binder and (w_eff_/c)—effective water-binder ratio, where w_eff_ is the total mass of water used in the concrete mix, minus the mass of water entrained in the modifier serving as the internal curing agent [6,39].

The problem of unambiguous determination of w/c affects the interpretation of published research results on the impact of SAP on strength parameters. In the vast majority, published results represent a reduction of compressive strength as the content of SAP in the concrete mix increases [20]. The biggest problem in determining the actual change of mentioned properties is to determine which of the two previously mentioned water-binder coefficients is comparative with the reference series: (w/c)_tot_ or (w/c)_eff_. In most studies, water intended for absorbtion by SAP is not included in the effective water-binder ratio. It is a result of adopting a similar methodology as with other internal curing agents. This leads to a situation where the assessment of the SAP influence on the properties of the concrete composite is carried out by modifying two parameters simultaneously: mass SAP content in the tested composition and additional water needed to fully activate the modifier. Such a methodology makes it difficult to draw correct conclusions as to what the real impact of SAP on the properties of concrete composites is. It can be argued that with the introduction of additional water to the concrete mix, without the use of SAP, an increase in the water-binder ratio is obtained. This results in the deterioration of mechanical properties and an increase of porosity of the concrete, leading to a reduction in shrinkage deformations. The introduction of additional water into the concrete mix will therefore affect the same parameters on which the influence of SAP is tested. The adoption of such a methodology leads to conclusions based on two phenomena occurring simultaneously and affecting the same properties of a concrete composite, making it impossible to properly determine the impact of SAP itself on the above-mentioned parameters.

The aim of the conducted research was to determine the impact of SAP material characteristics and an alternative to the commonly used method of dosing SAP to concrete mix on the effectiveness of internal curing. The percentage change in compressive strength and linear change caused by shrinkage deformations in relation to the reference series was assumed as a means to measure effectiveness. As part of the experiment, two types of SAP were used, differing in material characteristics and the potential to absorb water in different environments. After preliminary absorption tests of the used modifiers, ten series of specimens were made with differing mass content of used SAP and different dosing methodologies. Specimens were tested for compressive strength and linear changes caused by shrinkage deformations.

## 2. Materials and Methods 

### 2.1. Characteristic of Basic Materials and Testing Methods

The CEM I 42.5 R cement used for the preparation of the tested concrete mixes has been compliant with the requirements of EN 197 [40]. Mineral fine aggregate (Vistula River sand) and natural gravel aggregate (2/4, 4/8 and 8/16, mm) have been used for the preparation of concrete mixes in accordance with the requirements of EN 12620 standard [41]. In the preparation of the tested concrete composite, tap water was used in accordance with EN 1008 [42]. In order to increase the flowability of the tested concrete mixes a superplasticizer was used. It was characterized by a steric and electrostatic mechanism of action and a maximum content allowed by the manufacturer in the concrete mix at the level of 3% of the binder mass.

To study the influence of SAP on selected properties of a concrete composite, two types of SAP with different grain sizes and properties were used, as seen in Table 1. SAP M was characterized by average grain size of 150–850 µm and SAP D by 2.0–2.5 mm. Absorption potentials were set on the basis of preliminary tests under conditions reflecting the conditions of different methods of dosing the additive to the concrete mix.

The methodology of the research on the impact of SAP on the development of the linear changes caused by shrinkage deformations was carried out using the Amsler apparatus. It was conducted on prismatic specimens with a square cross-section of 100 × 100 mm and a length of 500 mm (Polish standard PN-84/B06714/23 [43]). The concrete mix was compacted in two layers on a vibrating table. The specimens were demoulded after one day and transferred to a climatic chamber with a constant temperature of 20 °C and relative air humidity exceeding 90%. Measurements of the course of the linear changes started with the demoulding of the specimens, i.e., one day after the preparation of the concrete mix. The specimens were then placed in a climatic chamber in which they stayed between successive markings of the linear changes. For the first two weeks measurements of linear changes were performed daily. For the next two weeks, the measurements took place twice a week. Subsequent measurements were made at the frequency of one measurement per week.

The methodology of the conducted research on the impact of SAP on compressive strength after 28 days was developed in accordance with EN-12390-3 [44] using 150 × 150 × 150 mm specimens cured in climatic chamber.

The consistence tests of the concrete mix were made using slump test according to EN 12350-2 [45].

### 2.2. Methods of Dosing SAP into a Concrete Mix

**Figure 2:**
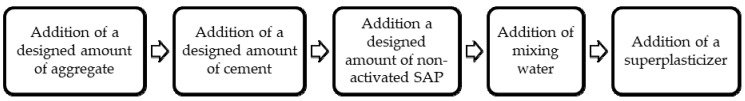
Method R: The order in which components were added to the concrete mix when using the methodology of adding non-activated superabsorbent polymers (SAP).

**Figure 3:**
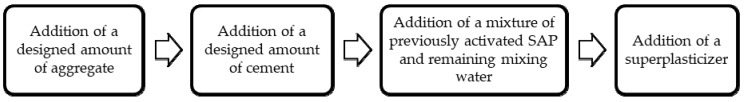
Method O: The order in which components were added to the concrete mix when using the methodology of adding activated superabsorbent polymers (SAP).

The conducted research included an assessment of the impact of the method of dosing SAP to the concrete mix on the formation of selected concrete composite parameters. Two dosing methods were included: by adding non-activated SAP to the other dry components of the concrete mix, as seen in Figure 2, method R, and by adding a mixture of activated SAP and mixing water to the remaining components, shown in Figure 3, method O. The main difference between the methodologies discussed is the use of a different course of water absorption and desorption in each case.

In the case of the method R, the absorption of the mixing water by the SAP takes place with the addition of water to the remaining components of the concrete mix, i.e., the activation of SAP takes place in a highly active electrochemical concrete mix environment. Due to the reversible nature of water absorption by SAP, its ability to absorb water strongly depends on the environment (tap water, cement paste, or concrete mix) in which this phenomenon occurs. In the concrete mix environment, the ability of SAP to absorb the mixing water is limited to 10–15% of the absorption achieved in the environment of tap water. This ultimately leads to a much lower intensity of curing, due to the relatively small amount of water being desorbed from the activated SAP particles during the hydration process.

Method O omits the stage of absorption of mixing water from a concrete mix and focuses on maximizing the desorption potential of previously absorbed tap water. Thanks to the prior activation of SAP in the tap water environment, a much higher initial degree of SAP saturation with water can be achieved, and thus it significantly increase the desorption potential in a highly electrochemically active environment. The time between preparing and adding the mixture of SAP and mixing water to the rest of components of the concrete mix depends on the material characteristics of tested SAP. In the case of SAP M mixture, it was added to the concrete mix within 5–10 min after activation. In the case of SAP D, 24 h were needed to achieve full SAP activation. Nonetheless, the authors’ observations suggest that the period between SAP activation and addition to the mix could be significantly longer, without compromising the impact of SAP on the concrete properties.

#### Absorption or Desorption—Ways to Achieve Equilibrium

**Figure 4:**
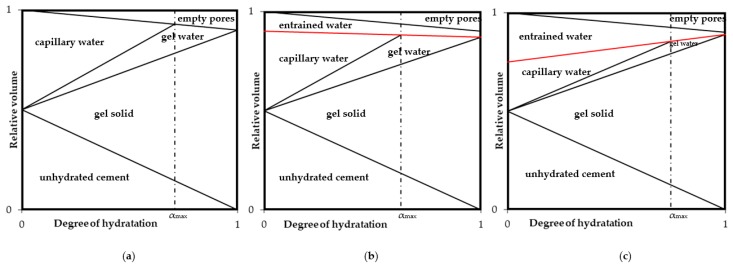
Powers model in three variants. (**a**) Sealed system, not modified with superabsorbent polymers; hydration takes place until the capillary water is exhausted [47]; (**b**) Sealed system, modified by superabsorbent polymers based on the dosing method assuming the addition of non-activated SAP to other dry components of the mix; (**c**) Sealed system, modified with superabsorbent polymers based on the dosing method involving the addition of a mixture of previously activated SAP and remaining mixing water to the rest of components of the concrete mix. A detailed description of charts in the text.

The hypothesis that prompted the authors to check the efficiency of the method O in comparison to the widely used method R was the idea of using differences in SAP absorption potentials in environments with different electrochemical activity to intensify the internal curing process. Assuming these differences, one can expect changes in the course of desorption of water from SAP activated in various environments. In order to illustrate hypothetical changes in the course of absorption and desorption of water during the hydration process, modified Powers charts were developed. These charts allow a schematic representation of changes in the phase distribution in the cement matrix during the course of hydration, as shown in Figure 4.

In the case of concrete with a specific water-cement ratio, the distribution of phases during hydration proceeds in accordance with the empirical model developed by Powers and Brownyard [46] in 1948, allowing the estimation of the volumetric composition of materials included in the cement matrix. This model allows classification of water contained in concrete into three groups: capillary water (free water), physically bound water (gel water) and chemically bound water (gel solid). In the case of Figure 4a, the external environment’s influence is limited due to the use of selected methods of external curing. The maximum degree of hydration is limited by the amount of capillary water needed to ensure the conditions for the course of hydration [47].

If the (w/c)_tot_ in the case of Figure 4b is kept at the same level, i.e., without introducing an additional volume of water into the concrete mix, the activation of SAP introduced into the concrete mix in the inactive state occurs at the expense of some of the mixing water. The process of water absorption from a concrete mix takes place along with the mixing of concrete mix components. Due to this, the maximum saturation of SAP before starting hydration can be assumed. The most common SAP dosing for concrete composites is at 0.3–0.6% of the binder [1] and the absorption capacity of SAP in the cement paste environment is in the range of 8–30 g/g [20]. This allows it to assume the amount of absorbed water by SAP at about 5–10% of the mixing water. This value may vary as it depends on the designed water-binder ratio. With such predefined initial conditions and the activation of the SAP in a strongly alkaline environment, the absorption capacity of SAP will be significantly impaired. Along with the increase of the degree of hydration, the desorption of water from SAP will occur. This SAP behaviour is caused by a loss of the balance between osmotic pressure of the water-saturated SAP and the hydrostatic pressure/internal stresses of the concrete. Assuming a reduced absorption potential in a highly electrochemically active environment and a continuous increase in osmotic pressure, accompanyied by the desorption of water from SAP, when the maximal hydration degree α_max_ is reached, the aforementioned balancewill be restored. This fact means entraining some of the absorbed water in the SAP structure even after achieving the maximum degree of hydration.

If in the case of Figure 4c, where the (w/c)_tot_ is kept the same as in option (b), SAP activation takes place at the expense of a portion of the mixing water. However, the absorption process takes place in the tap water environment. A mixture of activated SAP and remaing mixing water is added to the rest of components of the concrete mix. Assuming the same mass content of the applied SAP as in case (b), the increase in the percentage of absorbed mixing water is caused by the absorption potential of SAP in the tap water environment approximately 10 times higher than in the cement paste environment. With such defined initial conditions and using the full absorption potential of the SAP mass used from the moment of SAP introduction into the mix, only rapid desorption of water from the SAP will occur. It is caused not only by stress in the concrete, but also by a highly active electrochemical environment. This will contribute to significant reductions in linear changes in the initial phase of hydration. Considering the compressive strength development by entraining a significant fraction of the mixing water in the SAP’s structure during concrete hardening, the water migration beyond the concrete would decrease, thus the maximal degree of hydration would increase. By maintaining the porosity at a similar level as in the example unmodified with SAP, shown in Figure 4a, one can expect an increase in strength parameters.

### 2.3. Absorption Tests of SAP

**Figure 5:**
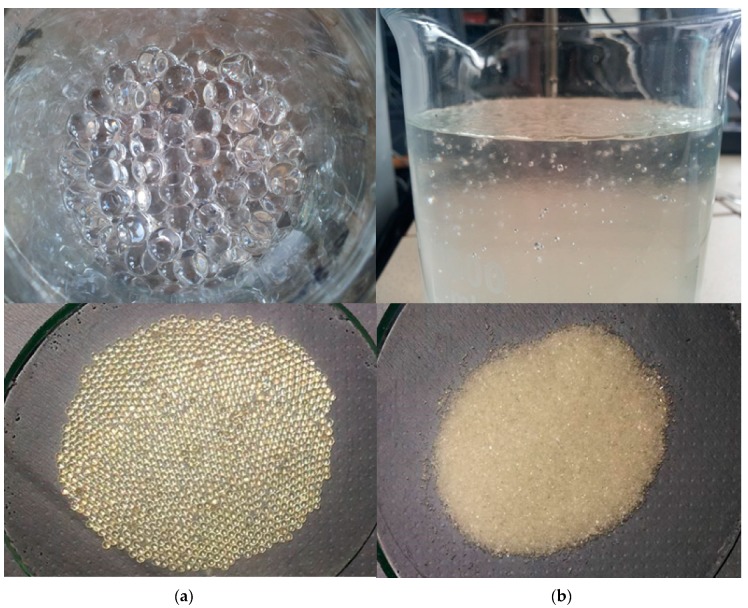
Differences in the water absorption mechanisms of two applied superabsorbent polymers (SAP): (**a**) activated SAP D on the top and non-actiovated in the bottom; (**b**) activated SAP M on the top and non-activated in the bottom.

The first step in designing compositions of concrete mixes tested in the research program was the determination of the absorption capacity of SAP used. The SAP used in the study, shown in Figure 5, had a different effect accompanying water absorption, resulting from differences in the grain size of SAP.

The absorption of water by the SAP M led to the formation of a homogeneous mixture of saturated SAP in a liquid state. It had increased viscosity in relation to the viscosity of tap water. However, the SAP D did not form a homogeneous mixture after saturation—it was possible to remove the excess of non-absorbed water from between the swollen grains of the SAP. In addition, at the stage of preparation for carrying out absorption tests, an observation was made regarding the mechanism of absorption of SAP M. It forced the application of absorption capacity assessment methods other than the one commonly used in the literature. SAP M, added to the tap water in a non-dispersed form, tended to rapidly absorb water at the interface between the water and SAP layers. This resulted in creating conglomerates consisting of an activated SAP layer on the outside and non-activated in the center from which the water supply was cut off by the saturated outer layer. This phenomenon made it impossible to correctly assess the absorption capacity on the basis of the teabag method test [20], consisting of placing unsaturated SAP in a sachet in a test solution. This forced the development of another methodology for the assessment of the above-mentioned properties of the SAP M, as shown in Table 2.

Due to the absorption mechanism of the SAP D, a standard teabag method test was carried out as part of the assessment of the SAPs absorption potential of tap water environment [20]. It reflected the absorption potential of SAP D with the assumed dosing method for concrete mixes—the addition of a mixture of previously saturated SAP and remaing mixing water to the rest of components of the concrete mix. Due to the long time necessary for a full activation of SAP D, the absorption assessment took place 48 h after the placement of the SAP in the tap water environment. Under the same conditions and for the same time, the SAP was activated before being added to the tested concrete mixes.

In the case of the SAP M, an alternative method for assessing the water absorption capacity had to be developed. Due to the different absorption mechanism, the teabag method was considered too inaccurate and not reflecting the conditions that characterized the tested methods of SAP dosing to the concrete mix. Instead of assessing the absorption potential of the SAP M itself, the assessment of the properties of the mixture of SAP and tap water was conducted. More specifically, a study of viscosity of the mixture depending on the mass of water to the mass of SAP ratio was conducted. This action was taken due to the assumed method of dosing the SAP M, i.e., by adding a mixture of SAP M and the mixing water to the dry components of the concrete mix. By observing the viscosity changes depending on the mass of SAP M to the water mass ratio, it was possible to estimate the practical absorption of the SAP, in conditions in which it was added to the concrete mix.

It was problematic to determine the absorption potential of the SAP M with the method of introducing unsaturated SAP into the concrete mix. The water absorption capacity of SAP in the concrete mix environment is strongly impaired in comparison with the absorption in the tap water environment. It is caused by the highly electrochemically active nature of such environment. In the literature, this problem is usually solved by preparing a solution of a cement paste with a water-cement ratio of 5–7 [20] or by preparing a mixture of ions present in cement paste in a water solution and carrying out the teabag method test [29]. It can be argued if determining the absorption potential in such artificially created solutions reflects the conditions occuring in the concrete mix environment. Due to complications in the preparation of such solutions and the predictable inaccuracy of such tests, in the method of adding unsaturated SAP to the dry components of the concrete mix it was decided to determine the absorption capacity of the SAP M based on the conclusion drawn from the literature analysis where the absorption potential in the environment set to simulate the conditions occuring in the concrete mix is usually about 10 times lower than the absorption potential in a tap water environment.

Due to the long activation time of the SAP D, no studies on the effect of the addition of the above-mentioned unsaturated SAP to the dry components of the concrete mix were made, since the determination of the actual absorption capacity under such dosing conditions would be extremely inaccurate.

The absorption of the SAP D was determined by performing the teabag method test, as seen in Table 3.

**Figure 6:**
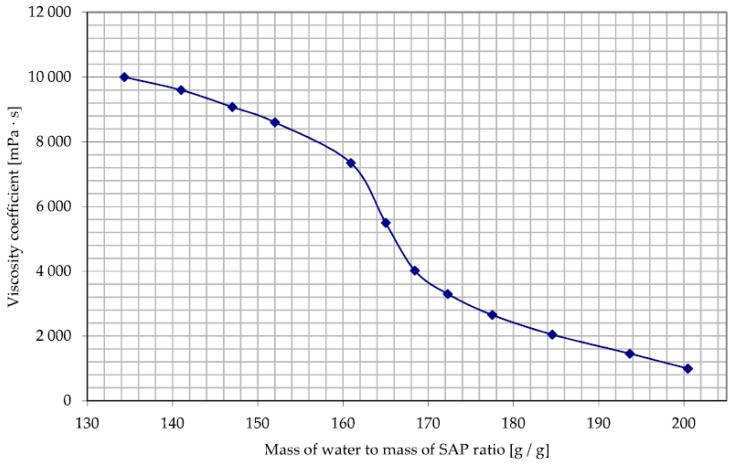
Chart showing the change in dynamic viscosity of the tap water mixture with superabsorbent polymers (SAP) M depending on the mass of water to mass of SAP M ratio.

The absorption potential of the SAP M in the tap water environment was determined based on the authors’ method of measuring the viscosity changes resulting from the change of the water to SAP M mass ratio in the mixture tested. The rapid drop in the viscosity of the mixture, visible in Figure 6 above the absorption capacity of 160 g/g can be interpreted as reaching the state of full saturation of the SAP M and the emergence of an increasing volume of ‘free water’, not absorbed by SAP and responsible for decreasing viscosity. The value of 160 g/g was taken as the absorption capacity of the SAP M in the tap water environment.

### 2.4. Compositions of Concrete Mixes

In order to investigate the effect of an alternative method of dosing SAP on the formation of concrete composite properties, ten concrete mix compositions were developed. Those included a reference series, not modified with SAP and nine SAP modified mixes. Reference series’ composition represented a concrete with low w/c ratio and with a substantial mass of the binder. It was designed that way in order to increase the contribution of the autogenous shrinkage to the total shrinkage of the concrete. Also, by composing binder solely from CEM I the influence of other mineral additions on the tested concrete properties was mitigated. Six of them were modified with the SAP M and three with SAP D. The developed compositions also differed in mass content of the SAP used. As part of the analysis developed on the basis of the results of laboratory tests, the percentage of the mixing water absorbed by the SAP in each of the tested series of specimens was considered as a comparative parameter.

All tested series of specimens were characterized by the same total water-cement ratio set at 0.3, the same aggregate composition and the same amount of binder used, as seen in Table 4. In addition, in order to be able to determine the effect of SAP and its dosing methods on the formation of the consistence of the concrete mix, the same amount of superplasticizer, representing 2.3% of the cement mass, was used in each of the tested series of specimens.

Series of specimens modified with SAP differed only in the quantitative SAP content in a given concrete composite and the method of dosing SAP into a concrete mix. A total of nine compositions have been prepared containing SAP M or SAP D (Table 5).

In each of the designed compositions the total water-cement ratio—(w/c)_tot_, was 0.3, so that it would be possible to compare the results obtained as a result of the research with the results of the reference series. The total water content in SAP modified mixes was always the same as in the reference mix, shown in Table 4. By maintaining (w/c)_tot_ on a constant level in all prepared series of specimens the only variables affecting changes in the properties of the tested concrete composites were the quantitative content of SAP in a given specimen series and the percentage of water absorbed by SAP.

Initially the influence of SAP D was to be tested in dosages allowing the absorption of 25, 50 and 75% of the mixing water successively. However, during the preparation of the SAP DO 50 mix, a significant decrease in consistence was observed. Based on this observation, it was assumed that the mix with 75% of the mixing water absorbed by the SAP D, will be extremely dry and it would impossible to compact it on the vibrating table. Due to this, a decision was made to change the dosages allowing the absorption of 12.5, 25 and 50% of the mixing water successively.

## 3. Results

Conducted experiments consisted of compressive strength tests, the study of linear changes caused by shrinkage deformations and tests of the consistence of concrete mixes. In addition to the research, a representation of the results of individual specimen series was developed, as part of the analysis of the results. It was prepared in reference to the reference series and a previously presented summary of published research on the range of reduction of linear changes. Aforementioned representation of the results was presented as a function of the absorbed percentage of mixing water, shown in Figure 1.

### 3.1. Consistence of Concrete Mixes

To determine the effect of the addition of SAP on the consistence of the concrete mixes, a slump test was performed, the results of which are presented in Table 6.

### 3.2. Compressive Strength

**Figure 7:**
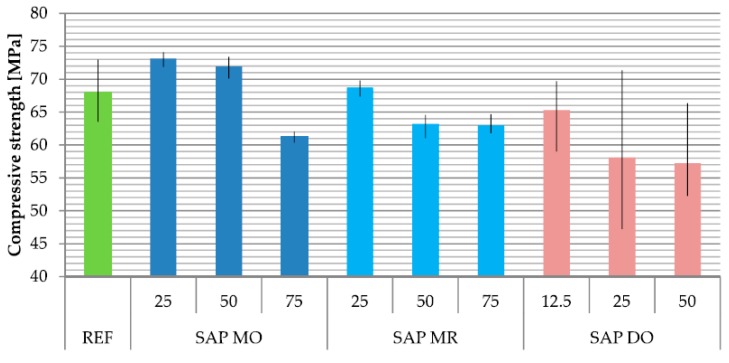
The average compressive strength of the tested compositions of concrete composites.

As part of the determination of the effect of the addition of SAP on average compressive strength, a destructive test was carried out in accordance with the EN-12390-3 standard. The results of this test are presented in Figure 7 and Table 7.

### 3.3. Linear Changes Caused by Shrinkage Deformations

**Figure 8:**
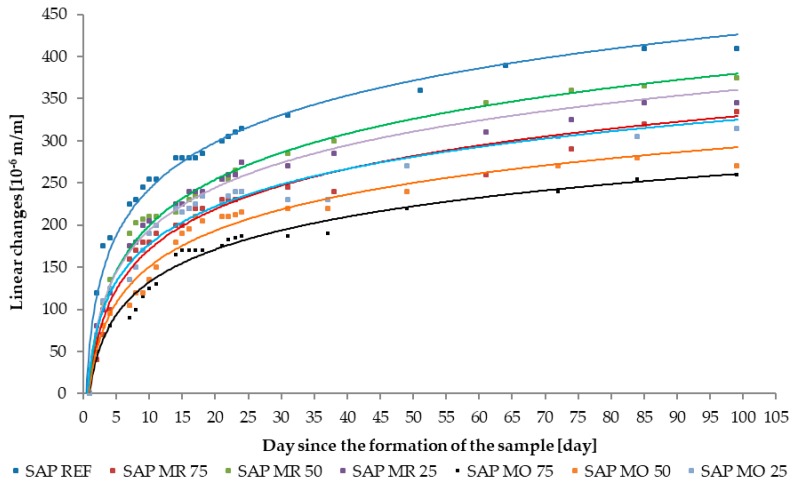
The effect of dosing method on the autogenous shrinkage of series modified with fine grade superabsorbent polymers (SAP), (SAP M). The linear changes axis presents shrinkage, not expansion.

**Figure 9:**
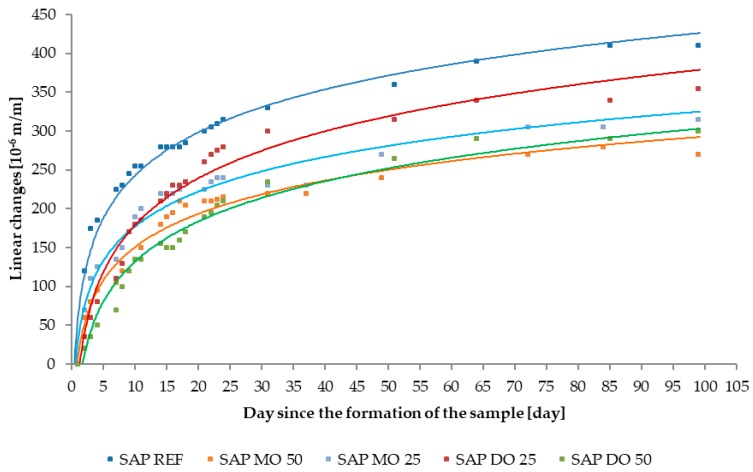
The effect of type of superabsorbent polymers (SAP) (SAP M and SAP D) on the autogenous shrinkage. The linear changes axis presents shrinkage, not expansion.

**Figure 10:**
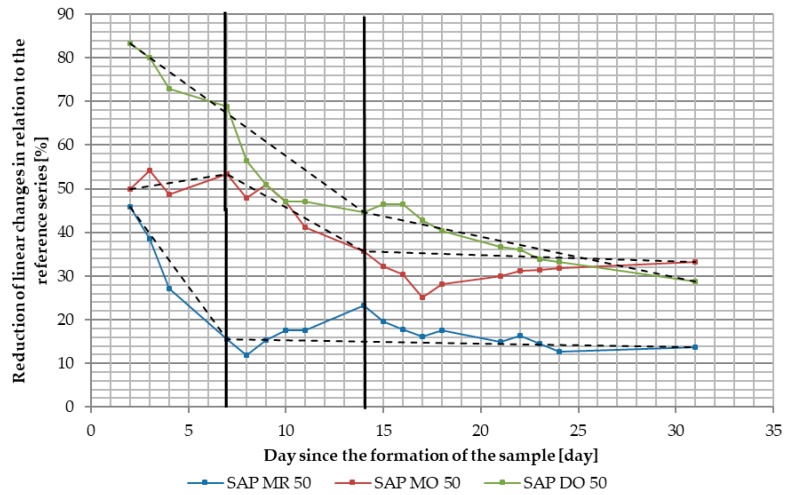
Chart showing the course of changes of the linear changes of tested specimen series of superabsorbent polymers (SAP) (SAP MR 50, SAP MO 50 and SAP DO 50) in relation to linear changes of the reference series ratio at each day of the first month of measurments.

In order to analyze the impact of SAP on the shaping of linear changes caused by shrinkage deformations it was decided to present the results of tests on two different types of graphs. The first of them, shown in Figure 8 and Figure 9, present the autogenous shrinkage of individual tested series of specimens using approximation by means of logarithmic curves. It allows determination of the effectiveness of SAP as an anti-shrinkage agent in concrete technology, as seen in Table 8. The second type of prepared graphs illustrate the partial changes between the series containing SAPs and the reference series for the first 31 days of measurements. This method of presenting research results, shown in Figure 10, allows to follow the course of shaping the linear changes of individual specimen series and to determine the activation time of SAP.

The second type of prepared graphs illustrates the partial changes between the series containing SAP and the reference series for the first 31 days of measurements. This method of presenting research results allows the following of the course of shaping the linear changes of individual specimen series and to determine the activation time of SAP, shown in Figure 10. The course of changes of the presented results was divided into three phazes. For the SAP DO 50 series, a rapid decrease in the reduction of linear changes occured within two weeks. It was followed by a steady decrease until setting at approximately 13% reduction in comparison with reference series after 99 days. In the case of SAP MO 50, within first week of measurments, a reduction in linear changes at about 50% of the reference series was maintained. Between the first and the second week, the reduction of the linear changes started to drop and eventually set on a level of 35–45%. It was maintained at this level until the end of the measurments. SAP MR 50 series represented a rapid decrease in shrinkage reduction within the first week, droping from approximately 45% in reduction to about 18% in a matter of days. The reduction of linear changes of was about 15% compared with reference series was then maintained until the end of measurments.

## 4. Discussion

All of the tested concretes containing SAP were characterized by showing smaller linear changes caused by shrinkage deformations than the reference concrete composite. The biggest reduction, reaching 43% after 31 days, was achieved by the SAP MO 75 mix.

**Figure 11:**
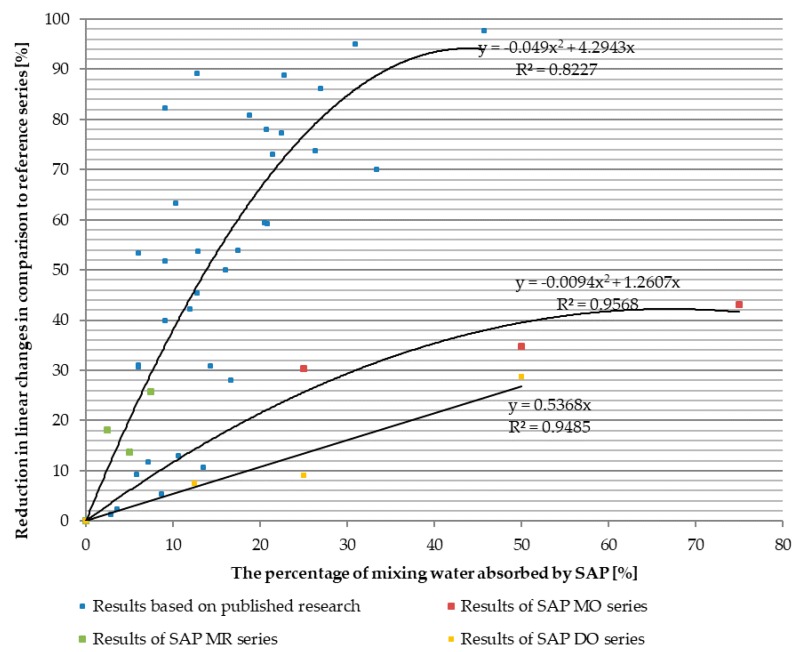
Relations between the reduction of the linear changes and the percentage of the mixing water absorbed by superabsorbent polymers (SAP) in the case of previously analyzed results of research carried out in the scientific literature, shown in Figure 1, and authors’ studies on series of SAP MO, SAP MR and SAP DO.

Taking into account the characteristics of the course of changes of the concrete series tested for linear changes, the obtained results, as seen in Figure 1, can be compared with the previously presented literature analysis. In the case of mixes modified with SAP M and prepared by adding the mixture of a previously activated SAP and the remainig mixing water (SAP MO series, Figure 11), a change can be observed in relation to the results of tests carried out in the literature [20,26,27,28,29]. The reason for these differences is the change of the dosing methodology in the conducted research program. In MO series, SAP was pre-saturated with water before adding it to the concrete mix. It allowed the full potential of SAP to absorb water, i.e., the absorption potential of the tested SAP was determined as the absorption potential in the tap water environment. On the contrary, in most studies carried out in the scientific literature [20,21,26,30] the absorption potential was determined in a strongly alkaline environment. The change of methodology led to a situation in which the lower mass content of the SAP used in the conducted research program was able to absorb about ten times more water than in the case of following the methodology present in the scientific literature [29,49]. The change in adopted absorption potential also resulted in a change in the mass content of SAP in the concrete mix in relation to the tests carried out in the literature. The most common dosages used in the literature methodology, which involved adding non-activated SAP to dry concrete mix components, are approx. 0.3–0.6% of cement mass [1,35]. However, in the case of studies on SAP MO series, the mass of SAP required to absorb 75% of the mixing water was only 0.114% of the cement mass.

In the case of mixes containing SAP MR dosed according to the method of adding non-activated SAP to the remaining dry components of the mix, one can see the confirmation of the correctness of the model used in the presentation of research results in the literature, seen in Figure 11. Despite the fact that mass dosing of SAP MR was the same as for the respective series of MO concrete mixes, a decrease in the amount of water absorbed by SAP M was observed. It was caused by the reduced absorption potential in a strongly alkaline environment [29], leading to a decrease in the efficiency of the reduction of linear changes caused by shrinkage deformations.

The results of the tests for linear changes caused by shrinkage deformations for SAP D modified mixes, where SAP D was added as the mixture of a previously activated SAP and the remainig mixing water, allowed to observe the influence of another variable on the internal curing. Although SAP D was added to the concrete mix in the same way as the SAP M in the MO series, it was characterized by a different grain size, absorption potential and different mechanism of water absorption. These differences resulted in a change in the efficiency of reduction of linear changes, seen in Figure 11. What is important to emphasise is that absorption capacity of SAP D in the tap water environment was significantly lower then absorption capacity of SAP M. In the case of SAP D it was 73.8 g/g compared with SAPs M 160 g/g. Along with the average grain size of 2.0–2.5 mm, far less swollen particles of SAP D were introduced to the concrete mix while most likely affected the rate and range of the internal curing.

It can be concluded that SAP DO series were characterized by the lowest reduction in linear changes caused by shrinkage deformations despite the higher mass content of SAP in the concrete mix—in the case of the SAP DO 50 series it was 0.204% of the binder mass. Despite the larger mass content of the SAP in the concrete mix, it obtained a smaller reduction in linear changes than concrete composites of the MO series. The mechanism of water absorption in the case of SAP D may be responsible for such a phenomenon. SAP D did not form a homogeneous mixture with the added mixing water, so the reduction of linear changes occurred locally. It resulted in a rapid desorption of absorbed water and led to reducing the final efficiency of linear change reduction.

The method of adding previoulsy activated SAP M to the concrete mix increased the compressive strength by almost 10% compared to the reference series. This increase could be observed in the case of absorption of 25–30% of the mixing water by SAP. This influence of SAP in the applied method of dosing is probably caused by several factors. The first of these factors is the even distribution of SAP in the structure of the cement matrix. Assuming such a behavior of the SAP M, the cement matrix structure is densified along with the release of the absorbed water—as the absorbed water is released evenly in the entire volume of the concrete composite, the global degree of hydration increases. 

Another factor allowing as explanation as to why the changes in compressive strength differ depending on the SAP dosing methods used is the mechanism of water absorption by the tested SAP samples. When a pre-saturated SAP is added to the dry components of the concrete mix, a homogeneous mixture of activated SAP and water is obtained. In the case of the addition of non-activated SAP to the concrete mix, SAP conglomerates may form [20], due to the strong electrochemical activity of the concrete mix. These conglomerates desorb mixing water and thus contribute only to increase in local hydrataion degree and the formation of a large quasi-pore networks after the desorption of water [20]. These factors are likely to cause a global decrease in the strength of concrete mixes made using the methodology of adding the non-activated SAP directly to the concrete mix. 

With the increase in the percentage of mixing water absorbed by SAP above 60% in the MO series of mixes the compressive strength of the material starts to deteriorate. It can therefore be concluded that the change of the methodology of adding SAP to the concrete mix allows modification of the influence of SAP on the compressive strength only in a given range of dosages. Beyond this range there is an almost linear decrease in strength characteristic. 

Also, the change from effective water-cement ratio mainly reffered to in literature [47] to total water-cement ratio as a comperative parameter with the reference series may have influenced the analysis of the results. Without introducing an additional volume of water to the concrete mix, the number of possible explanations for changes in compressive strength drops significantly. With only SAP being added to the concrete mix, an increase in compressive strength can be explained in two ways. Either the degree of hydration increases slightly without cardinally compromising the pore distribution or the opposite effect takes place—the degree of hydration increases significantly and is accompanied by slight disturbance in pore phase distribution. In the presented paper, SAP dosages were between 0.05–0.2% of the binder mass. In comparison, the dosages in most of published reseach are usually several times higher [1,20]. Also, the variation of the results of compressive strength tests was reduced in series containing SAP M compared with reference series. Those two facts seem to indicate that the homogeneity of the material increased, i.e., the degree of hydration increased slightly without compromising the pore distribution. This conclusion stands in stark contrast with other published results [27,37,38,47]. Usually with higher dosages of SAP introduced into concrete mix by means of the most common methodology of dosing, a decrease in mechanical properties of concrete is observed [32,33]. It is most likely that the increase in the mass content of the non-activated internal curing agent causes a densification of cement matrix and appearance of defects in its structure simultaneously. 

This conclusion would suggest that a different approach to internal concrete curing with SAP investigated in this paper could mitigate SAP’s negative effects on the pore phase distribution in hardened concrete. However, as aforementioned mitigation is reached by lowering the dosage of SAP in the mix, the anti-shrinkage efficiency of the modifier drops as well. The reach of this mitigation should be investigated further by studying the actual pore phase distribution in microstructure of concretes, modified with SAP, with different material characteristics and using different dosing methods. 

The analysis of compressive strength results of SAP MR and SAP MO series, seen in Table 7, showed the decrease of variation of results in comparison with reference series. This may suggest an increase of homogeneity of the material. The opposite effect is observed in the case of course grade SAP (SAP DO series).

In the case of SAP MR series of concrete mixes, results fit in the trend of literature research results [27,32,33,38]. The drop in strength is increasingly stronger as the percentage of mixing water absorbed by SAP increases.

In the case of the tested series of SAP DO specimens, one can observe a decrease of the compressive strength as the percentage of mixing water absorbed by SAP increases. The reason for the decrease in strength can be explained by the increase in the porosity of the concrete composite after the desorption of the SAP characterized by a considerable grain size. SAP D probably contributes to the formation of a large pore network, which despite the local increase in cement hydration degree at a direct proximity from the SAP particles, have a negative impact on the heterogeneity of tested concrete composites.

### Determination of Optimal SAP Content and Dosing Methods

**Figure 12:**
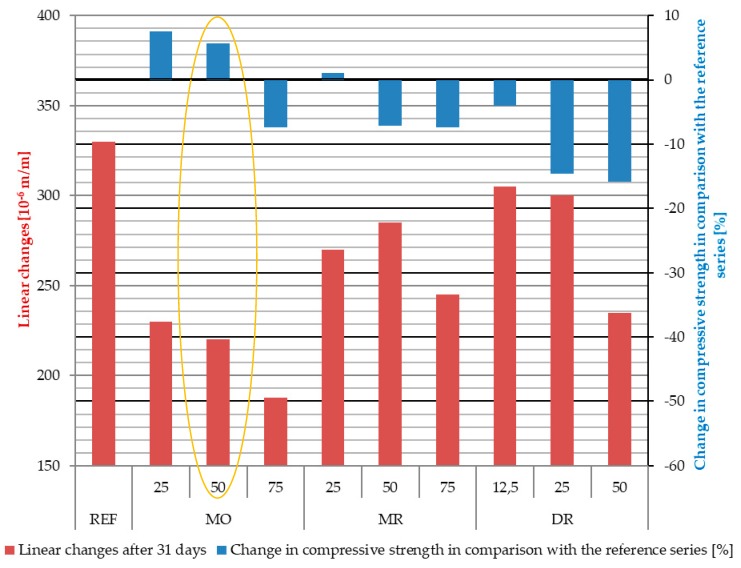
Changes in the compressive strength of superabsorbent polymers (SAP) modified series in comparison with the reference series and the effect on linear changes caused by shrinkage deformations. The optimal composition is marked with the ellipse.

In order to determine the optimal composition and the method of dosing SAP, a graph, seen in Figure 12, was developed to show changes in compressive strength of modified SAP concrete composites in relation to the reference concrete composite and impact on linear changes caused by shrinkage deformations. The assumption accompanying the determination of the optimal composition of the concrete mix, shown in Figure 12, was to achieve the highest possible reduction in linear changes while maintaining or increasing compressive strength in relation to the reference series. Of all tested concrete mixes, only three were characterized by an increase in strength compared to the reference series—SAP MO 25 series (7.51%), SAP MO 50 (5.73%) and SAP MR 25 (1.05%).

SAP is used as an additives in order to reduce linear changes caused by shrinkage deformations, therefore the optimal composition of a concrete mix modified with SAP was defined as the composition contributing to the greatest reduction of linear changes while maintaining or increasing the compressive strength in relation to a reference series. With such assumptions, the concrete composite of SAP MO 50, characterized by the largest reduction of linear changes and increased compressive strength in relation to the reference series, was considered an optimal composition of the concrete mix tested in the research program.

## 5. Conclusions

On the basis of conducted research on the effectiveness of internal curing with the use of SAP with different material characteristics, in different dosages, and using different methods of its introduction into a concrete mix with a composition typical for concretes in which the internal curing is recommended and applied, the following conclusions were made:

- The pre-saturation in tap water environment of SAP with fine graining allowed an increase in the efficiency of internal curing. In the case of SAP M with a small grain size compared to the same mass of SAP M applied to the concrete mix as non-activated, the increase of both the reduction of linear changes and the compressive strength compared to the reference series was observed.
- Regardless of the type of SAP used, its mass quantity and the amount of absorbed mixing water resulting from the dosing method, the SAP addition to the concrete mix reduced the linear changes of the concrete, especially in the first 7 days.
- Increase or lack of decrease in compressive strength as a result of modifying the concrete composite with pre-saturated SAP M depended on the amount of water absorbed by the SAP.
- The increase of SAP content in a concrete mix caused a decrease in the liquidity of the concrete mix, regardless of the type of SAP used, its mass quantity and the amount of the mixing water absorbed resulting from the dosing method.
- The use of SAP D particles characterized by the non-activated grain size of 2.0–2.5 mm, even if pre-saturated with tap water, resulted in a significant decrease in compressive strength and the significantly higher variation of the test results than in the case of SAP M modified compositions or reference series. This suggests an increase in material heterogeneity.
- The highest reduction in linear changes caused by shrinkage deformations in series modified with SAP occurred during the first week of measerments, potentially increasing the durability of the concrete at an early phase of concrete hardening, i.e., reducing cracking risk in first days after demoulding.

## Figures and Tables

**Table 1:** Material characterization of the superabsorbent polymers (SAP) used in research program according to the manufacturer’s data.

Characteristic		SAP M	SAP D
**Granulation**	**<150 µm**	≤8	0
	**150–850 µm**	86–95	0
	**>850 µm**	≤6	0
	**2.0–2.5 mm**	0	100
**Chemical characterization**		*Potassium acrylate and acrylic acid polymer*	*2-Propenoic acid, polymer with sodium 2-propenoate (1:1)*
**Solubility in water**		Insoluble	
**The polymerization process in SAP production**		Gel polimerization	Inverse suspention polimerization
**Particle shape**		Irregular	Spherical

**Table 2:** Tests carried out to determine the absorption potential of superabsorbent polymers (SAP) to absorb water depending on the SAP dosing method to concrete mixes.

Dosing Method	SAP ID	
	SAP M	SAP D
Method O	Assessment of SAP water absorption on the basis of the viscosity change test	Assessment of SAP water absorption on the basis of the teabag method test
Method R	Theoretical absorption at the level of 10% of SAP absorption potential in the tap water environment	-

**Table 3:** Absorption potential of superabsorbent polymers (SAP) D in tap water environment based on the teabag method test.

SAP ID	Mass of Unsaturated SAP (g)	SAP mass after 48 h Saturation with Tap Water (g)	Absorption of SAP (g/g)
SAP D	1.09	80.44	73.8

**Table 4:** Composition of the reference concrete mix (SAP REF).

Material	Weight per 1 m^3^/kg
Vistula sand 0/2 mm	668
Gravel 2/4 mm	95
Gravel 4/8 mm	477
Gravel 8/16 mm	668
CEM I 42.5 R	450
Tap water	135
Superplasticizer	10.35

**Table 5:** List of prepared superabsorbent polymers (SAP)-modified concrete mixes. Each of the mixes characterized by the same total water-cement ratio (w/c)_tot_ = 0.3.

Series ID	SAP ID	Dosing Method	SAP Content in Relation to Cement Mass (%)	The Percentage of Mixing Water Absorbed by SAP (%)
SAP MO 25	SAP M	Method O	0.047	25
SAP MO 50	SAP M		0.094	50
SAP MO 75	SAP M		0.141	75
SAP MR 25	SAP M	Method R	0.047	2.5^1^
SAP MR 50	SAP M		0.094	5^1^
SAP MR 75	SAP M		0.141	7.5^1^
SAP DO 12.5	SAP D	Method O	0.051	12.5
SAP DO 25	SAP D		0.102	25
SAP DO 50	SAP D		0.204	50

^1^ The theoretical percentage of mixing water absorbed by SAP based on literature analysis.

**Table 6:** The results of a slump test performed on concrete mixes modified with superabsorbent polymers (SAP) and on the reference series.

Series ID	Slump Test According to EN 12350-2 [45] (cm)	Class According to EN 206 [48]
SAP REF	6.0	S2
SAP MO 25	6.0	S2
SAP MO 50	3.5	S1
SAP MO 75	3.0	S1
SAP MR 25	6.0	S2
SAP MR 50	4.5	S1
SAP MR 75	2.0	S1
SAP DO 12,5	3.0	S1
SAP DO 25	1.5	S1
SAP DO 50	0	S1

**Table 7:** Average compressive strength of the tested compositions of concrete composites and the difference between superabsorbent polymers (SAP) modified concrete series and reference series.

Series ID	Compressive Strength		Change in Average Compressive Strength in Relation to the Reference Series (%)
	Average Value (MPa)	Variation (%)	
SAP REF	68.0	5.6	0.00
SAP MO 25	73.1	1.2	7.51
SAP MO 50	71.9	1.8	5.73
SAP MO 75	61.2	1.25	−10.05
SAP MR 25	68.7	1.4	1.05
SAP MR 50	63.2	2.4	−7.08
SAP MR 75	63.0	1.9	−7.43
SAP DO 12.5	65.3	6.9	−4.06
SAP DO 25	58.1	17.1	−14.65
SAP DO 75	57.2	11.2	−15.89

**Table 8:** Percentage reduction in average linear changes in relation to the reference series on day 99 from specimen formation.

Series ID	Linear Changes after 99 Days (10^−6^ m/m)	Reduction in Linear Changes in Relation to the Reference Series (%)
SAP REF	410	0.00
SAP MO 25	315	23.17
SAP MO 50	270	34.15
SAP MO 75	260	36.59
SAP MR 25	345	15.85
SAP MR 50	375	8.54
SAP MR 75	335	18.29
SAP DO 12.5	395	3.66
SAP DO 25	355	13.42
SAP DO 75	300	26.83
